# Hexane extract from *Spondias tuberosa* (Anacardiaceae) leaves has antioxidant activity and is an anti-*Candida* agent by causing mitochondrial and lysosomal damages

**DOI:** 10.1186/s12906-018-2350-2

**Published:** 2018-10-19

**Authors:** Bruna Maria Pereira da Costa Cordeiro, Nataly Diniz de Lima Santos, Magda Rhayanny Assunção Ferreira, Larissa Cardoso Corrêa de Araújo, Alexsander Rodrigues Carvalho Junior, Alan Diego da Conceição Santos, Ana Paula de Oliveira, Alexandre Gomes da Silva, Emerson Peter da Silva Falcão, Maria Tereza dos Santos Correia, Jackson Roberto Guedes da Silva Almeida, Luís Cláudio Nascimento da Silva, Luiz Alberto Lira Soares, Thiago Henrique Napoleão, Márcia Vanusa da Silva, Patrícia Maria Guedes Paiva

**Affiliations:** 1 Departamento de Bioquímica, Centro de Biociências, Universidade Federal de Pernambuco, Recife, Pernambuco 50670-420 Brazil; 2 Departamento de Ciências Farmacêuticas, Centro de Ciências da Saúde, Universidade Federal de Pernambuco, Recife, Pernambuco 50740-520 Brazil; 3 Universidade CEUMA, São Luís, Maranhão 65075-120 Brazil; 4 Núcleo de Estudos e Pesquisas de Plantas Medicinais, Universidade Federal do Vale do São Francisco, Petrolina, Pernambuco 56304-205 Brazil; 5 Departamento de Antibióticos, Centro de Biociências, Universidade Federal de Pernambuco, Recife, Pernambuco 50670-420 Brazil; 6 Núcleo de Bioprospecção da Caatinga, Instituto Nacional do Semiárido, Campina Grande, Paraíba 58429-970 Brazil; 7 Núcleo de Nutrição, Centro Acadêmico de Vitória, Universidade Federal de Pernambuco, Vitória de Santo Antão, Pernambuco 55608-680 Brazil

**Keywords:** *Spondias tuberosa*, Hyperoside, Gallic acid, Fatty acids, Antifungal activity

## Abstract

**Background:**

*Spondias tuberosa* is a plant that produces a fruit crop with high economic relevance at Brazilian Caatinga. Its roots and leaves are used in folk medicine.

**Methods:**

Chemical composition of a hexane extract from *S. tuberosa* leaves was evaluated by thin-layer chromatography (TLC), high-performance liquid chromatography (HPLC) and ^1^H nuclear magnetic resonance (NMR). Antioxidant potential was investigated by DPPH and ABTS assays. Antifungal action on *Candida* species was evaluated determining the minimal inhibitory concentration (MIC_50_) and putative mechanisms were determined by flow cytometry analysis. In addition, hemolytic activity on human erythrocytes was assessed and the concentration required to promote 50% hemolysis (EC_50_) was determined.

**Results:**

Phytochemical analysis by TLC showed the presence of flavonoids, hydrolysable tannins, saponins and terpenes. The HPLC profile of the extract suggested the presence of gallic acid (0.28 ± 0.01 g%) and hyperoside (1.27 ± 0.01 g%). The representative ^1^H NMR spectrum showed saturated and unsaturated fatty acids among the main components. The extract showed weak and moderate antioxidant activity in DPPH (IC_50_: 234.00 μg/mL) and ABTS (IC_50_: 123.33 μg/mL) assays, respectively. It was able to inhibit the growth of *C. albicans* and *C. glabrata* with MIC_50_ of 2.0 and 0.078 mg/mL, respectively. The treatment of *C. glabrata* cells with the extract increased levels of mitochondrial superoxide anion, caused hyperpolarization of mitochondrial membrane, and compromised the lysosomal membrane. Weak hemolytic activity (EC_50_: 740.8 μg/mL) was detected.

**Conclusion:**

The results demonstrate the pharmacological potential of the extract as antioxidant and antifungal agent, aggregating biotechnological value to this plant and stimulating its conservation.

## Background

Brazil has one of the world highest levels of plant diversity and the north and northeast regions of the country concentrate much of this diversity. In the semi-arid region of northeastern Brazil (known as Caatinga) there are several kinds of plants employed in popular culture for the treatment of human diseases. In spite of the great diversity of Caatinga, there are still few studies on the potential of bioactive compounds coming from the plants of this region [1–3].

*Spondias tuberosa* Arruda, popularly known as “umbuzeiro” or “imbuzeiro”, is an endemic plant of Caatinga, adapted to survive and produce fruits even under hydrical and salt stress [4]. Several medicinal properties of *S. tuberosa* have been described, including treatment of digestive disorders, diabetes, menstrual cramps, diarrhea, inflammation of the kidneys, bacterial infections, and foot pain [5–7]. Tannins and flavonoids are found in the bark [8] and the fruits contain anthocyanins, ascorbic acid, minerals, flavonoids and carotenoids [9]. The high tannin content and the presence of natural antioxidants give to *S. tuberosa* fruits a functional appeal [10]. Hydroethanolic extract of leaves from *S. tuberosa* containing chlorogenic acid, caffeic acid, rutin and isoquercitrin demonstrated anti-inflammatory action [11]. Methanolic extract of the leaves showed in vitro activity against several strains of Gram-negative bacteria [2].

The chemical characterization of plant extracts allows the identification of chemical markers and suggests potential bioactivities that can be investigated. For example, flavonoids are secondary metabolites that have great pharmacological importance, since they act in the prevention of degenerative diseases. Among the biological properties described for these compounds, it can be highlighted cytotoxic, thrombolytic, anti-inflammatory, antitumor, vasorelaxant and antioxidant activities [11–13]. Other phenolic compounds such as tannins and anthocyanins are also considered important antioxidant agents. Natural antioxidants play an important role in health care as they provide protection from oxidative stress and associated diseases [14].

Natural products have also been considered an important source of bioactive compounds against infectious diseases [11]. Fungal infections have increased gradually over the last 30 years, becoming one of the more relevant public health problems. *Candida* yeasts are among the main etiological agents of invasive fungal infections, which are responsible for high mortality and morbidity rates throughout the world [15–17]. These fungi have developed resistance mechanisms against antibiotics, favoring the persistence and progression of the infection even when antifungal therapy is adequately performed [18]. *Candida albicans*, *Candida krusei*, *Candida parapsilosis* and *Candida glabrata* are among the most prevalent causers of candidiasis [19, 20].

In this context, this work reports the chemical composition of a hexane extract from leaves of *S. tuberosa* as well as the evaluation of the antioxidant potential and antifungal action on *Candida* species. Mechanisms involved in the antifungal activity against the most sensitive species were investigated. In addition, hemolytic activity was assessed as safety parameter to determine whether the extract would be able to damage erythrocytes membrane.

## Methods

### Materials

Leaves of *S. tuberosa* were collected at the *Parque Nacional do Catimbau* (Coordinates 08°37′23“ S, 37°09’21” W), Buíque, Pernambuco. The plant material was identified by Dr. Alexandre Gomes da Silva, and a voucher specimen was deposited at the *Instituto Agronômico de Pernambuco*, Recife, Brazil, under the reference number 91,090. Plant collection was authorized (number 16806) by the *Instituto Chico Mendes de Conservação da Biodiversidade* (ICMBio) from Brazilian Ministry of Environment. The access was recorded (A1503A6) in the *Sistema Nacional de Gestão do Patrimônio Genético e do Conhecimento Tradicional Associado* (SisGen).

Stored cultures of *Candida albicans* (URM 5901, from ungual scales), *Candida parapsilosis* (URM 6951, clinical isolate), *Candida glabrata* (URM 4246, from blood of AIDS patient), and *Candida krusei* (URM 6391, from human blood) were obtained from the Culture Collection of the University Recife Mycologia (URM), *Departamento de Micologia*, *Universidade Federal de Pernambuco* (UFPE).

### Extract preparation

The leaves were dried in a forced air convection oven at 45 °C until constant weight. After drying, they were powdered using Willye-type mill (model TE650; Tecnal, Brazil) and the powder was stored protected from light and moisture at 28 °C until use. The extract was prepared in a Soxhlet apparatus using 100 g of the powdered leaves and 1 L of *n*-hexane. The solvent was evaporated at 75 rpm and 64.4 °C in a HB10 rotary-evaporator (IKA Works, Wilmington, NC, USA). The resulting material after solvent evaporation corresponded to the extract.

### Thin-layer chromatography

An amount of 1 mg of the extract was dissolved in 1 mL of methanol. The sample was taken to the ultrasound for 30 min for complete solubilization. All standards (Table 1) were used at the concentration of 1 mg/mL in methanol. The sample and standards were applied manually on silica gel 60-F254 chromatography plates (Macherey-Nagel, Germany) and the different mobile phases and chromogenic agents used are shown in Table 1 [21–23]. The following secondary metabolites classes were investigated: steroids, flavonoids (aglycones and heterosides), cinnamic derivatives, mono-, tri- and sesquiterpenes, alkaloids and coumarins.

**Table 1:** Elution systems, chromogenic agents, and standards used in the phytochemical analysis of the hexane extract from *Spondias tuberosa* leaves with thin-layer chromatography (TLC)

Classes	Mobile phase	Chromogenic agent	Standards
Polyphenols (Hydrolysable tannins)	90:5:5	NEU + PEG	Gallic acid and Ellagic acid (Sigma-Aldrich, USA)
Condensed tannins	90:5:5	Chloridric vanillin	Catechin (Sigma-Aldrich, USA)
Flavonoids	90:5:5	NEU + PEG	Quercetin and Rutin (Sigma-Aldrich, USA)
Cinnamic derivatives	90:5:5	NEU + PEG	Caffeic acid and Chlorogenic acid (Sigma-Aldrich, USA)
Terpenes and steroids	70:30	Lieberman-Burchard + Δ	β-sitosterol (Sigma-Aldrich, USA)
Coumarins	50:50:50	KOH + Δ	Coumarin (Phytolab, Brazil)
Saponins	100:11:11:26	Lieberman-Burchard + Δ	Escin (Sigma-Aldrich, USA)
Reducing sugars	50:20:10:10	Thymol + H_2_SO_4_ 10% + Δ	D-fructose (ChromaDex, USA)
Alkaloids	50:6.75:5	Dragendorf	Pilocarpine nitrate (Sigma-Aldrich, USA)
Anthraquinones	50:6.75:5	HNO_3_ + KOH 10%	Sennoside A (Sigma-Aldrich, USA)

Systems: 90:5:5 – ethyl acetate: formic acid: water; 70:30 – toluene: acetate; 50:50:50 – ethyl eter: ethyl acetate: 10% acetic acid (saturation); 100:11:11:26 – ethyl acetate: acetic acid: formic acid: water; 50:20:10:10 – ethyl acetate: acetic acid: formic acid: water; 50:6.75:5 – ethyl acetate; methanol; water. *NEU* Neu’s reagent. *PEG* polyethylene glycol

### High-performance liquid chromatography coupled to diode array detector (HPLC-DAD) analysis

The extract was analyzed by high-performance liquid chromatography (HPLC) in an Ultimate 3000 coupled to a diode array detector (DAD) and equipped with a binary pump (HPG-3x00RS), degasser and automatic sampler with a loop of 20 μL (ACC-3000). All these equipments were from Thermo Fisher Scientific (USA). An amount of 0.25 g of the hexane extract was weighed, transferred to a 25-mL volumetric flask and diluted with methanol. The solution was taken to the sonicator during 15 min to complete solubilization (stock solution). After solubilization, an aliquot (1 mL) of the stock solution was transferred to a 10-mL volumetric flask and the volume was completed with ultrapure water (Purelab^®^, Elga LabWater, USA). Hyperoside (HWI Analytic Gmb, Germany) and gallic acid (Sigma-Aldrich, USA) solutions (1 mg/mL in methanol) were used as standards. The sample solution and standard solutions were filtered (0.45 μm PVDF membrane). The chromatography was performed in a Dionex® C_18_ column (250 mm × 4.6 mm d.i., 5 μm) equipped with a Phenomenex® C_18_ pre-column (4 mm × 3.9 μm) at 26 °C. The wavelengths were set at 270 and 350 nm for detection of hydrolysable tannins and flavonoids, respectively. The mobile phase was composed by ultrapure water (A) and methanol (B), both acidified with 0.05% (*v*/v) trifluoroacetic acid, and the flow rate was adjusted to 0.8 mL/min. The following gradient program was used: 0–10 min, 5–20% B; 10–13.5 min, 20–25% B; 13.5–18 min, 25–40% B; 18–25 min, 40–80% B; 25–30 min, 80% B; 30–34 min, 80–5% B; 34–36 min, 5% B. The data were analyzed and processed using the software Chromeleon 6.8 (Dionex, Thermo Fisher Scientific, USA). The peaks of substances in the hexane extract were identified by comparing the retention times, UV spectra and subsequently, by spiking the sample with a small amount of the standards. The contents of hyperoside and gallic acid in the extract were calculated based on calibration curve obtained by chromatography of each standard at different concentrations: hyperoside – *y* = 3.8841*x* – 11.663 (R^2^ = 0.9987); gallic acid – *y* = 1.2097*x* – 0.9732 (R^2^ = 0.9996). The results were expressed as mean ± standard deviation.

### Nuclear magnetic resonance (NMR) experiments

One-dimensional and two-dimensional nuclear magnetic resonance (NMR) experiments were acquired in deuterated dimethylsulfoxide (DMSO-d_6_), using NMR Bruker AVANCE III 400 spectrometer, operating at 9.4 Tesla, observing ^1^H and ^13^C nuclei at 400 and 100 MHz, respectively. The spectrometer was equipped with 5-mm multinuclear direct detection probe (BBO). Due to the low sample concentration and the presence of water in the deuterated solvent, ^1^H NMR spectra were acquired through *zg* (classical pulse sequence) and *zgpr* (for water signal suppression). Besides, one-bond heteronuclear (^1^H-^13^C) and homonuclear (^1^H-^1^H) correlations were assessed by HSQC and COSY experiments. ^1^H and ^13^C NMR chemical shifts are given in ppm referenced to TMSP-*d*_*4*_ signal at 0.00 ppm.

### Antioxidant activity

#### Free radical scavenging activity by DPPH (2,2-diphenyl-1-picrylhydrazyl) assay

Antioxidant activity of the hexane extract was evaluated by the DPPH scavenging assay according to Mascato et al. [24], with some modifications. The hexane extract was diluted in methanol to reach concentrations ranging from 15.625 to 1000 μg/mL. In each assay, 270 μL of the DPPH solution (23.6 μg/mL in methanol, prepared on the day of the analysis) was added to the sample (30 μL). Methanol (30 μL) was used in the negative control. After 30 min in the dark, the reduction of DPPH was determined by measuring the colorimetric change at 517 nm. Ascorbic acid (0.5, 1, 2, 3 and 4 μg/mL, in ethanol) was used as standard. The antioxidant concentration required to decrease 50% of the DPPH present (IC_50_) was determined by exponential regression analysis. Two independent experiments were performed in triplicate. Samples were classified according to the IC_50_ as follows: IC_50_ < 50 μg/mL, very strong antioxidant; 50 < IC_50_ < 100 μg/mL, strong antioxidant, 101 < IC_50_ < 150 μg/mL, moderate antioxidant; IC_50_ > 150 μg/mL, weak antioxidant [25].

#### ABTS [2,2′-azino-bis(3-ethylbenzothiazoline-6-sulfonic acid] assay

The radical ABTS^+^ was generated by oxidation of an ABTS solution (7 mM) with 2.45 mM potassium persulfate solution. The mixture was allowed to react for 12 h in the dark at 25 °C before use. For the test, the ABTS^+^ stock solution (1 mL) was diluted in 60 mL of methanol to obtain an absorbance of 0.70 ± 0.02 at 734 nm. Next, 2.7 mL of this solution was added to 0.3 mL of 0.5, 1.0, 2.0, 3.0, 4.0 and 5.0 μg/mL Trolox® (standard) solutions prepared in methanol or 31.25, 62.5, 125, 250, 500 and 1000 μg/mL of extract. The absorbance was taken 6 min after the adding of the radical at 734 nm. The test was performed in triplicate. The extract concentration required to decrease 50% of the ABTS^+^ content (IC_50_) was determined by linear regression. Samples were classified according to the IC_50_ as described in the previous section.

### Antifungal assay

The yeasts were cultured in Sabouraud Dextrose Broth (SDB) at 28 °C for 16 h under gentle shaking. Next, the optical density at 600 nm (OD_600_) of the cultures was adjusted in order to correspond to 3 × 10^6^ CFU/mL. In each row of a 96-well microplate, 100 μL of the hexane extract (8.0 mg/mL) were serially diluted (1:1) in SDB and 20 μL of yeast culture were added to each well. Wells containing only the culture medium were used as sterility control while the 100% growth control contained the microorganism in culture medium. In addition, it was performed a blank composed by the extract diluted in culture medium. The OD_600_ was recorded at time zero and after incubation at 28 °C for 24 h. The increase in OD_600_ was considered as fungal growth. The minimal inhibitory concentration (MIC_50_) corresponded to the lowest extract concentration able to promote a reduction higher or equal to 50% in growth. Each assay was achieved in triplicate and three independent experiments were performed.

The supernatant (10 μL) from each well containing the extract at concentration higher or equal to the MIC_50_ was transferred to Sabouraud-Dextrose Agar plates and incubated for 24 h. The minimal fungicidal concentration (MFC) corresponded to the lowest extract concentration able to reduce the number of CFU in 99.9%. Each assay was carried out in triplicate in three independent experiments.

### Flow cytometry analysis

Fluorescent probes were used in order to analyze putative effects of the extract on mitochondrial superoxide production, the mitochondrial membrane potential, and lysosomal membrane of *C. glabrata* cells. In all these assays, yeast cells were resuspended at a density of 1 × 10^6^ cells/mL in RPMI-1640 medium supplemented with MOPS. The extract was added at the concentrations of 2 × MIC (0.156 mg/mL) or 4 × MIC (0.312 mg/mL), and the cells were incubated at 28 °C.

For measurement of mitochondrial superoxide production, the extract was added and, after 1 h, the medium was removed, and the cells were washed using PBS. Then the MitoSOX Red mitochondrial superoxide indicator (Molecular Probes, Invitrogen, Carslabad, CA, USA) at 5 μM was added and the samples were incubated for 10 min at 37 °C, protected from light. The cells were washed with warm buffer (three times) and analyzed by flow cytometry (FL3 channel, Accuri™, BD Biosciences, San Jose, CA, USA).

In other assays, cells were incubated with extract for 12 h and then washed with PBS and stained with 1 μg/mL acridine orange (lysosomal dye) in the dark for 20 min or 10 μg/mL rhodamine 123 (to assay mitochondrial membrane potential change) in the dark for 10 min. After the incubation, yeast cells were washed and analyzed by flow cytometry (FL3 channel and FL1 channel for acridine orange and rhodamine 123. respectively). A minimum of 10,000 events were analyzed in each condition. Changes in the fluorescent intensity of rhodamine 123 were quantified using the variation index (VI) obtained by the eq. (MT-MC)/MC, where MC is the mean of fluorescent intensity of control and MT the mean of treated cells. Negative values of VI correspond to membrane depolarization of mitochondria, while positive values indicate membrane hyperpolarization.

### Hemolytic assay

The hexane extract was evaluated for hemolytic activity in 96-well microplates according to Costa-Lotufo et al. [26]. Each well received 100 μL of a 0.85% (*w*/*v*) NaCl solution containing 10 mM CaCl_2_. Next, samples (100 μL) of the extract at 10 to 2500 μg/mL were added to the wells. Finally, each well received 100 μL of a 2% (*v*/v) suspension of human erythrocytes in 0.85% saline containing 10 mM CaCl_2_. In negative control, 150 μL of the saline solution plus 50 μL of 5% (v/v) DMSO were plated. Positive control (to obtain 100% hemolysis) contained 20 μL of 0.1% (v/v) Triton X-100 in 180 μL of saline solution. After incubation for 1 h at 27 °C and centrifugation, the supernatant was discarded, and the amount of released hemoglobin was measured by absorbance at 540 nm. Three independent experiments were performed in triplicate. Extract was considered active whether the EC_50_ value was lower than 200 μg/mL [26].

### Statistical analysis

Standard deviations (SD) were calculated using GraphPad Prism version 4.0 for Windows (GraphPad Software, San Diego, California, USA) and data were expressed as a mean of replicates ± SD. Significant differences between treatment groups were analysed by Student’s *t*-test (significance at *p* < 0.05) using Origin 6.0 program.

## Results

**Fig. 1:**
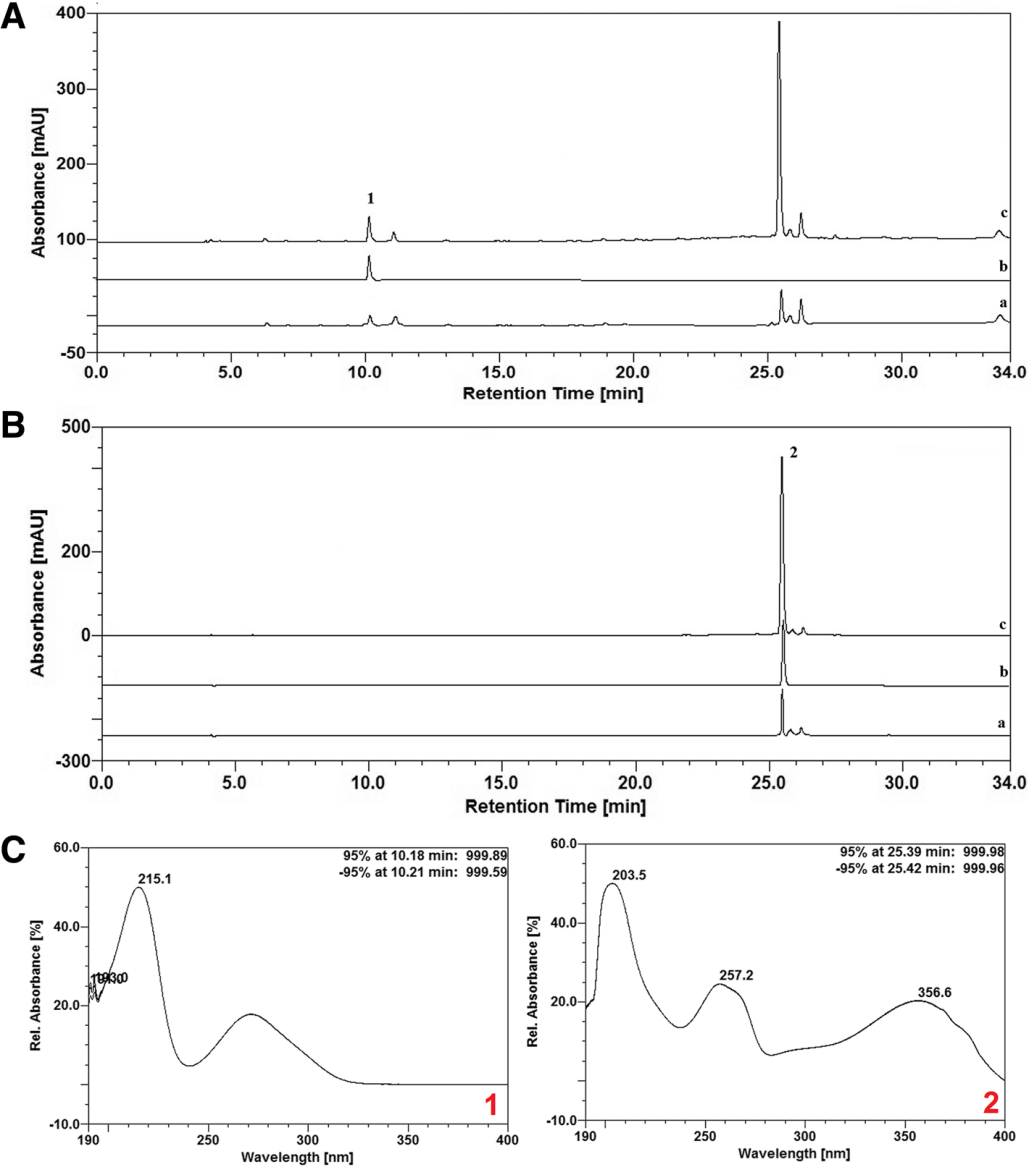
HPLC-DAD analysis of hexane extract from *Spondias tuberosa* leaf. Chromatographic fingerprint at 270 (**a**) and 350 (**b**) nm showed the presence of gallic acid (1) and hyperoside (2), as indicated by the retention times. **c** Scanning spectra of peaks 1 and 2

The phytochemical analysis by TLC of the hexane extract from *S. tuberosa* leaves showed the presence of flavonoids, hydrolysable tannins, saponins and terpenes. The HPLC profile of the extract at 270 nm (Fig. 1A, a) showed three main peaks (retention times 10.197, 25.403 and 26.223 min). The first peak probably corresponded to gallic acid (peak 1), in comparison with retention time of the standard (10.23 min; Fig. 1A, b). The chromatogram at 350 nm (Fig. 1B, a) showed main a peak at 25.433 min, which corresponds to a flavonoid (peak 2) since it was also observed at 270 nm. According to the retention time of the standard hyperoside (Fig. 1B, b), this peak is similar to this compound. These results were confirmed by the increase in the area after spiking the extract with the standards gallic acid (Fig. 1A, c) and hyperoside (Fig. 1B, c). The equivalent contents of hyperoside and gallic acid calculated were 1.27 ± 0.01 g% and 0.28 ± 0.01 g%, respectively.

**Fig. 2:**
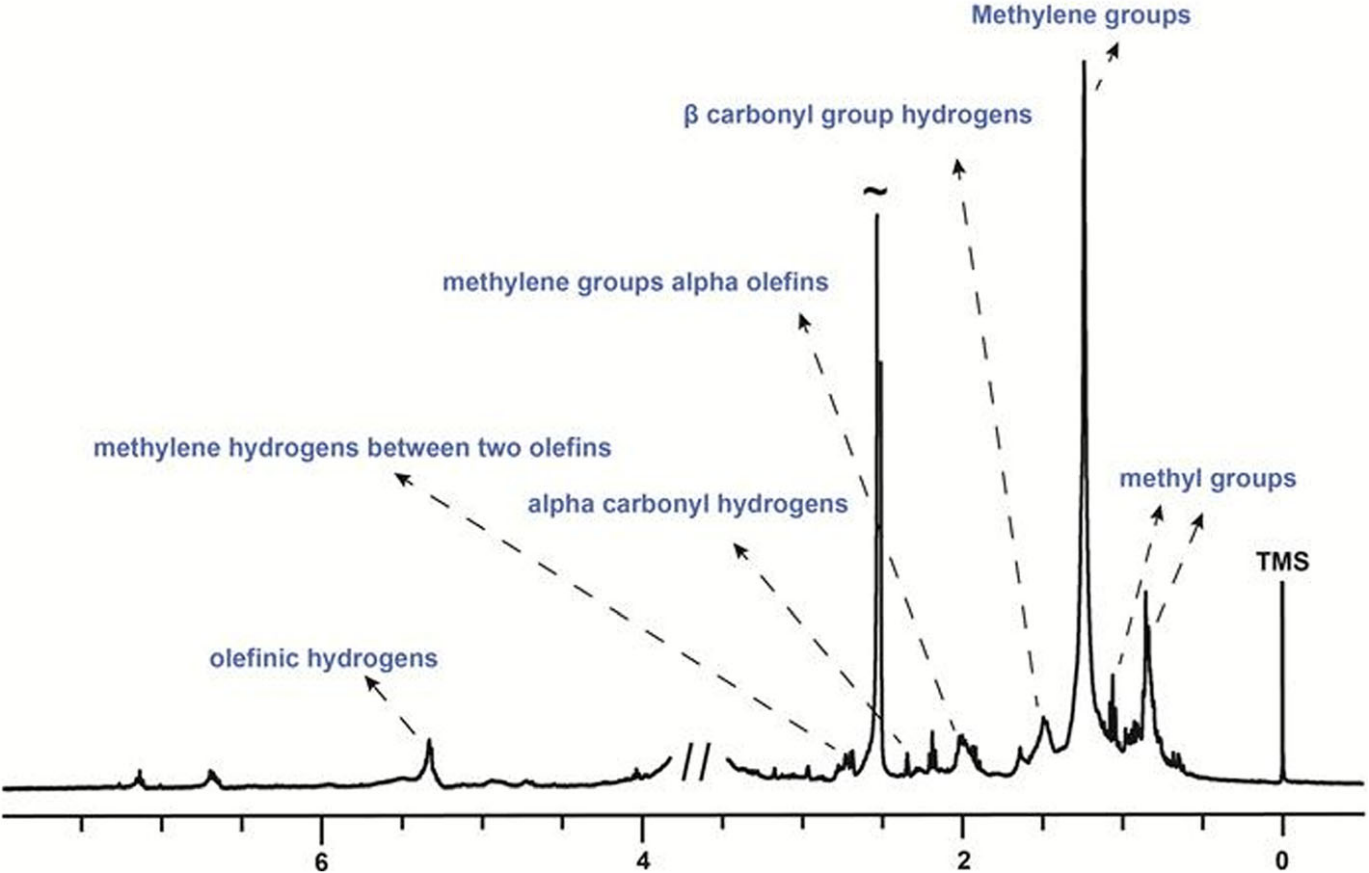
Representative ^1^H NMR spectrum of hexane extract of *Spondias tuberosa* leaf

The representative ^1^H NMR spectrum of hexane extract of *S. tuberosa* leaves revealed typical signals of fatty acid methyl esters (Fig. 2), which is expected for an extract obtained using a non-polar solvent. The resonances related to aliphatic hydrogens of all fatty acids can also be visualized in the figure. Methylene groups appeared at 1.16–1.35 ppm, while the signal at 1.49 ppm corresponds to β carbonyl group hydrogens. Characteristic resonances of methylene alpha-olefins of all unsaturated fatty acids were observed at 1.98 ppm. The presence of methylene hydrogens between two olefins and alpha carbonyl hydrogens could be noticed at 2.77 and 2.34 ppm, respectively. The signal at 0.84 ppm corresponds to methyl groups of all fatty acids, except for the methylic hydrogens of *n*-3 polyunsaturated acyl groups that are easily identified as a triplet at 1.06 ppm [27, 28]. It should be pointed out that the fatty acid acyl chains are not esterified to glycerol backbone, as typical signals of it were not observed. Another way of corroborating such information would be by checking the signal around 3.67 ppm, which is associated with the methoxy group of fatty acid methyl esters. However, in our experiment the residual water signal compromised the obtainment of such information. Finally, the signal at 5.33 ppm (olefinic hydrogens) connected to the carbon at 129.5 ppm indicated the presence of unsaturated fatty acids. The presence of hydrogens of phenolic compounds could be observed by signals between 6 and 8 ppm.

The hexane extract was evaluated for antioxidant activity by the DPPH scavenging assay. The results showed that the increase in the extract concentration led to higher inhibition of this free radical. An IC_50_ value of 234 μg/mL was calculated. The results from ABTS assay showed that the IC_50_ values were 123.33 and 1.72 μg/mL for the hexane extract and the positive control Trolox®, respectively.

The antifungal assays showed that the hexane extract was able to inhibit the growth of *C. albicans* and *C. glabrata* with MIC_50_ values of 2.0 and 0.078 mg/mL, respectively. *Candida parapsilosis* and *C. krusei* did not have their growth affected by the extract. Fungicidal effect was not detected.

**Fig. 3:**
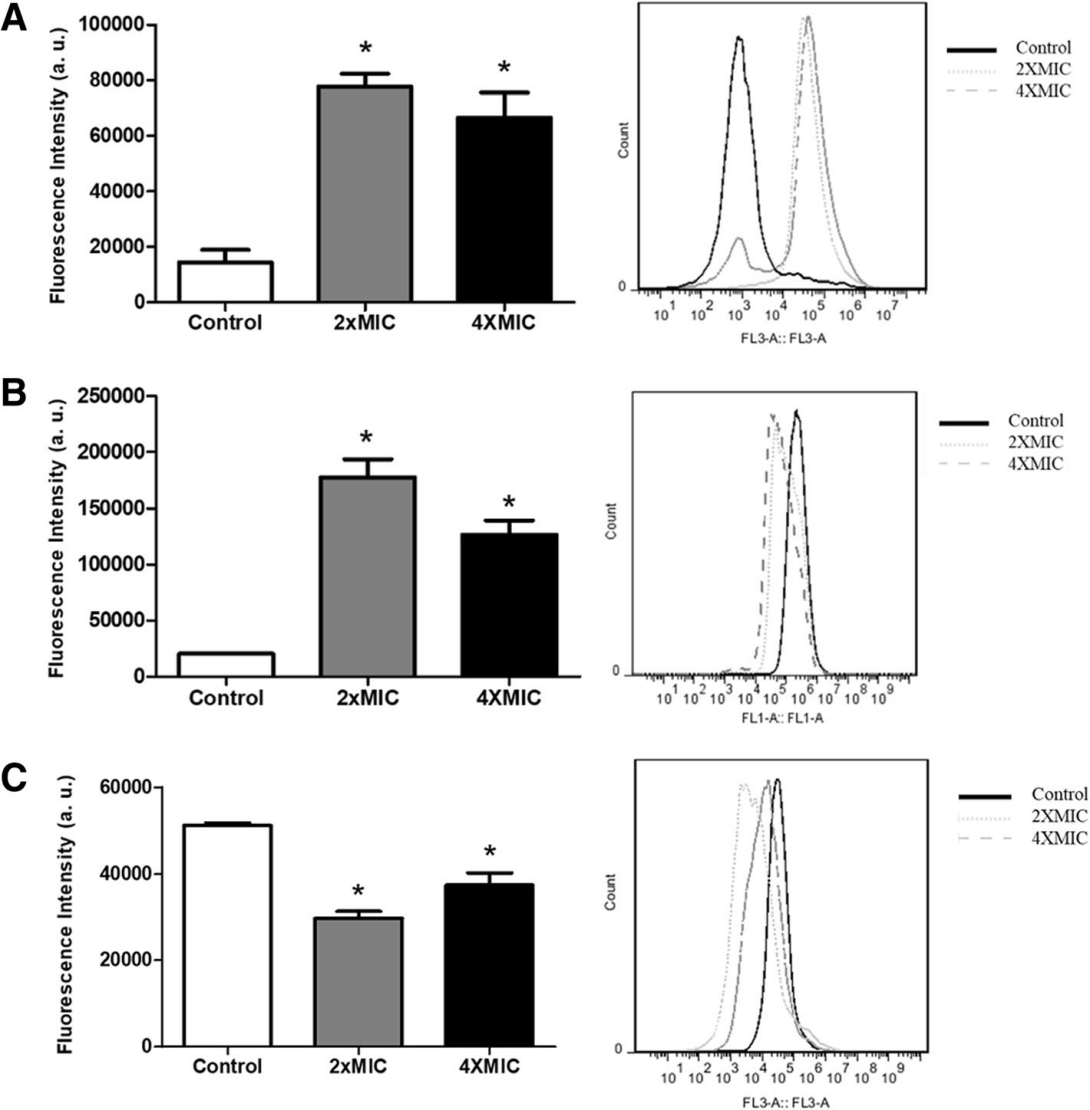
Investigation of antifungal mechanisms of hexane extract from *Spondias tuberosa* leaf against *Candida glabrata*. It was evaluated the effects of extract treatment on the production of mitochondrial superoxide anion (**a**), mitochondrial membrane potential (**b**) lysosomal membrane stability (**c**). (*) Significant differences compared with untreated cells (control). Minimal inhibitory concentration (MIC_50_) was 0.078 mg/mL

The effect of the extract on superoxide production by *C. glabrata* cells was evaluated. The results (Fig. 3A) showed that low levels of fluorescence were detected in untreated cells while high levels of mitochondrial superoxide anion were induced by treatment with the extract for 1 h (5.8 and 5.2 folds for 2× MIC_50_ and 4× MIC_50_, respectively, when compared with untreated yeasts). No statistical differences were observed between the amount of superoxide induced by the two concentrations tested.

Alterations in mitochondrial functions induced by the extract were evaluated by measuring changes the mitochondrial membrane potential (ΔΨm) using the variation index (VI). Healthy cells incorporated the probe rhodamine 123 and showed high levels of fluorescence emission. The fluorescence intensity was significantly increased by extract treatment when compared with untreated cells (Fig. 3B), resulting in positive VI values (7.65 folds for 2× MIC_50_ and 5.15 folds for 4× MIC_50_).

The lysosomal function was analyzed using an acridine orange-based assay. As expected, the yeast cells that were not treated with the extract exhibited a strong fluorescence emission, confirming that these cells had intact lysosomes. On the other hand, the extract treatment compromised the lysosomal membrane of *C. glabrata*, as seen by the lower levels of fluorescence emission than control cells (Fig. 3C). The fluorescence emission reduced 41.98% and 26.91% when the cells were incubated with the extract at 2× MIC_50_ and 4× MIC_50_, respectively.

The hexane extract was also evaluated for hemolytic activity against human erythrocytes. The EC_50_ value determined was 740.8 ± 61.09 μg/mL.

## Discussion

Many works have studied the chemical composition and biological activities of extracts from *S. tuberosa* tissues, including the leaves, obtained using polar organic solvents. For example, methanolic extract from *S. tuberosa* leaves contained phenols, tannins, flavones, flavonoids, leucoanthocyanidins and saponins [2] and Uchôa et al. [29] reported that extracts from the leaves of *S. tuberosa* in methanol and ethyl acetate were rich in flavonoids and triterpenes as well as also contained cinnamic derivatives. Analysis of a hydroethanolic extract from leaves of this plant also evidenced the presence of flavonoids [11]. In the present work, we aimed to evaluate the composition of an extract from *S. tuberosa* leaves obtained with hexane, a non-polar solvent. Interestingly, the classes of compounds detected by TLC were also present in the extracts mentioned above, produced using solvents with higher polarity. Particularly, the presence of flavonoids and hydrolysable tannins in the hexane extract caught our attention and stimulated a more in-depth evaluation by HPLC analysis. Indeed, the presence of compounds similar to gallic acid and hyperoside was confirmed.

Siqueira et al. [11] showed through HPLC-DAD analysis that a hydroethanolic extract of *S. tuberosa* leaves presented a large number of phenolic compounds and derivatives of flavonoids; they identified the presence of chlorogenic acid, caffeic acid, rutin, and isoquercitrin. Silva et al. [2] identified the presence of rutin, quercetin and ellagic acid in the methanolic extract of *S. tuberosa* leaves. These compounds were not detected in the hexane extract of *S. tuberosa* leaves described herein, which is probably due to the chemical characteristic of the hexane, which did not favor the extraction of high content of polar compounds. In spite of this, a specific flavonoid (probably hyperoside or a very similar compound) was efficiently extracted from the leaves using this solvent. The hyperoside is the 3-*O*-galactoside of quercetin and has been described in the literature as anticancer, anti-inflammatory, and antioxidant agent. In addition, this compound was shown to act in the protection of liver fibrosis and prevention of memory deficit [30–32].

According to the classification of Fidrianny et al. [25], the hexane extract of *S. tuberosa* leaves is considered a weak DPPH scavenger. However, the hexane extract from *S. tuberosa* leaves showed higher DPPH scavenging activity than extracts of *Spondias pinnata*, which showed IC_50_ ranging from 0.73 to 0.59 mg/mL [33]. The hyperoside possesses DPPH scavenging activity with an IC_50_ of 27.5 mM [34] and polyunsaturated fatty acids may act as antioxidants [35]. Thus, flavonoids and fatty acids may be involved in the antioxidant property of the hexane extract of *S. tuberosa* leaves.

According the results from ABTS assay, the extract is a moderate antioxidant. Floegel et al. [36] reported that high-pigmented and hydrophilic antioxidants in a variety of foods were better reflected by the ABTS assay in comparison with the DPPH assay. Indeed, the hexane extract of *S. tuberosa* leaves used in this work is highly pigmented and one of its main compounds is similar to hyperoside, a water-soluble compound. This may explain the better result obtained with ABTS in comparison with DPPH assay. The antioxidant properties of hyperoside have been demonstrated; for example, it protected endothelial cells against oxidative damage by hydrogen peroxide [37].

The hexane extract showed an interesting activity against *C. glabrata*, as seen by its low MIC_50_ value. Dall’Agnol et al. [38] reported that crude extracts of *Hypericum* containing the hyperoside showed no activity against yeasts. On the other hand, polyunsaturated fatty acids have demonstrated antifungal properties against *Candida* species [39].

Given the highest activity of the hexane extract against *C. glabrata* (as seen by its low MIC_50_ value), we attempted to analyze some subcellular alterations induced by this extract. The results demonstrate that the extract induced the production of mitochondrial superoxide anion and hyperpolarization of mitochondrial membrane. In addition, the extract damaged the lysosomal membrane of *C. glabrata* cells. Lysosomal membrane damage leads to release of cathepsins from into the cytosol where they participate in apoptosis signaling [40].

According to Costa-Lotufo et al. [26], a sample must present EC_50_ < 200 μg/mL to be considered hemolytic. Thus, the hexane extract from *S. tuberosa* leaves is considered no hemolytic, which is initial evidence to determine its safety.

## Conclusion

A hexane extract from *S. tuberosa* leaves containing flavonoids, hydrolysable tannins, saponins, terpenes and unsaturated fatty acids showed moderate antioxidant activity and antifungal effect on *C. glabrata*. Antifungal mechanisms against *C. glabrata* include increase in levels of mitochondrial superoxide anion, hyperpolarization of mitochondrial membrane, and damage to the lysosomal membrane. The extract showed weak hemolytic activity, which is important information for future studies to be performed at in vivo conditions. The results demonstrate the pharmacological potential of the extract, aggregating biotechnological value to this plant and stimulating its conservation.

## Abbreviations

ABTS: 2,2′-azino-bis(3-ethylbenzothiazoline-6-sulfonic) acid
CFU: Colony-forming units
DMSO: Dimethylsulfoxide
DPPH: 2,2-diphenyl-1-picrylhydrazyl
EC_50_: Effective concentration required to promote 50% hemolysis
HPLC-DAD: High-performance liquid chromatography coupled to diode array detector analysis
IC_50_: concentration required to decrease 50% of the free radical content
ICMBio: *Instituto Chico Mendes de Conservação da Biodiversidade*
MFC: Minimal fungicidal concentration
MIC_50_: Minimal concentration required to inhibit growth in at least 50%
NEU: Neu’s reagent
NMR: Nuclear magnetic resonance
OD: Optical density
PBS: Phosphate-buffered saline
PEG: Polyethylene glycol
SDB: Sabouraud Dextrose Broth
SisGen: *Sistema Nacional de Gestão do Patrimônio Genético e do Conhecimento Tradicional Associado*
TLC: Thin-layer chromatography
UFPE: *Universidade Federal de Pernambuco*
URM: University Recife Mycologia (culture collection)
VI: Variation index
